# Boundary violations and university teachers’ well-being during mandatory telework: Recovery’s role and gender differences

**DOI:** 10.1186/s12889-024-18178-6

**Published:** 2024-03-08

**Authors:** Madalena Mascarenhas, Vânia Sofia Carvalho, Cleide Fátima Moretto, Maria José Chambel

**Affiliations:** 1 Faculdade de Psicologia, CICPSI, Universidade de Lisboa, Alameda da Universidade, 1649-013 Lisboa, Portugal; 2 Universidade de Passo Fundo, 99052-900 Passo Fundo, Brasil

**Keywords:** Recovery, Psychological detachment, Boundary violations, Well-being, Flourishing, Gender equality, Telework, Work-family interface

## Abstract

**Background:**

This study aimed to explore the role of psychological detachment from work in the relationship of boundary violations and flourishing, as well as gender differences among university teachers during mandatory telework. We developed and tested a moderate mediation model where psychological detachment was the explanatory mechanism of the relationship between boundary violations with flourishing and using gender as the moderating variable.

**Methods:**

A cross-sectional study was conducted with a sample of 921 Brazilian university teachers (mean age 44 years, 681 women and 240 men) during mandatory telework. Multigroup analysis and moderate mediation were performed using Mplus 7.2.

**Results:**

Psychological detachment mediated the relationship between boundary violations (in both directions) and flourishing and work-to-family violations were more harmful to women’ recovery instead family-to-work violations were more harmful to men’ recovery, among university teachers during mandatory telework.

**Conclusion:**

By focusing on boundary violations in the context of mandatory telework, the study sheds light on the impact of blurred boundaries between work and personal life. This contributes both literature on work-life balance and literature recovery. Moreover, it helps to understand a crisis setting of remote work. Further, the study’s findings regarding gender differences highlight how men and women may experience and cope with boundary violations differently during mandatory telework, supporting future specific interventions across genders.

## Background

Telework is a flexible form of work that promotes the articulation between work and family, its imposition caused by the COVID-19 pandemic, has had negative consequences on this interaction and on well-being [1–7]. Although university teachers already carried out some tasks from home (e.g., preparing lessons or correcting tests), the lockdown forced them to carry out all educational activities remotely (e.g., lessons and meetings with colleagues or parents), without having the time and resources to prepare to do it [8, 9]. It was a major challenge that required effort from these professionals, as teaching in a distant format requires a range of information technology and different pedagogical skills that university teachers may not have (e.g., tech proficiency; to provide feedback in an online environment; active learning activities) [10]. It is also important to mention that the social distancing during the lockdown had a negative psychosocial impact, namely a reduction in opportunities for leisure activities [11].

According to boundary theory [12, 13] there are boundaries that separate different domains (e.g., work and family). Telework makes these boundaries more permeable and transitions (i.e., sudden role changes) are more frequent [12, 14–17]. Boundary violations are a specific form of transition, and it are characterized by the unwanted intrusion of one domain into the other, which consumes resources (cognitive and temporal), during or after the interruption [18, 19]. These violations can occur from work-to-family (e.g., answer a phone call from the boss after shift work) or family-to-work (e.g., help a child perform schoolwork during shift work) and they increased exponentially in the context of mandatory telework during COVID-19 with a negative impact on the well-being [12, 20–23]. The mandatory lockdown did not affect men and women equally: women lost more jobs, reduced, or fragmented their working hours and had more disruptions due to an increase in childcare and other domestic responsibilities (e.g., housework or eldercare) [24–27].

It is known that recovery, in special the psychological detachment from work, was greatly impaired by telework [28], and boundary violations were one of the most harmful aspects, as they decreased the time and availability required for recovery. All workers need to recover, which means, to replenish their resources, by psychologically distancing themselves from work (i.e., avoiding work-related thoughts or behaviours, and engaging in leisure activities) [29–32]. For recovery to occur, it is necessary to ensure that people do not engage in work-related activities in their free time [33]. According to the stressor-detachment model [34], work stressors do not allow the employees to distance from work when they are not working, and this happens because stressors trigger a highly negative response (e.g., ruminant thoughts or actions to decrease stressor) which don’t allow the person to psychological detach from the stressor. Boundary violations can be stressors because they are unscheduled interruptions that incur costs for the person, and they decrease the chances of recovery (either because they take time or because they keep work/family mentally present) [17, 35, 36]. For this reason, we intend to understand how boundary violations compromised the recovery of university teachers in lockdown, especially because university teachers have great difficulty in recovering [37, 38]. Furthermore, psychological detachment from work is important for well-being and flourishing [39, 40]. Flourishing is an indicator of subjective well-being and it is characterized by a positive emotional state, a high satisfaction with life and it is the major manifestation of mental health [41–43]. Boundary violations are harmful to flourishing because they imply the consumption of resources necessary for flourishing and cause negative emotions [20, 44–46]. We may understand why psychological detachment has this positive effect, as it is a protective factor against the negative effects of work stressors and decreases ruminant thoughts about work [34, 39], and, according to the stressor-detachment model, psychological detachment from work mediates the relationship between work stressors and well-being [34].

Thus, in the present study, it is expected to find a mediation whereas boundary violations (in both directions) will inhibit distancing from work, which, in turn, will hinder the flourishing of telecommuting university teachers during the period of confinement due to COVID-19.

Going beyond, we expect that the relationship between boundary violations and psychological detachment may be different between men and women. According to role theory [47], a person devotes more resources to the role that is most relevant to him or her, investing more effort and resources in maintaining the boundaries of that domain [47, 48]. Specifically, the family role is more salient in highly feminine people (i.e., the traits often associated with the female gender) and is tendentially more valued by women, while men tend to value the work role more [47, 48]. The fact that women have more family responsibilities and housework than men means that women should have more family-to-work violations [49] and telework women should have less effective recovery than men [50]. During mandatory telework, women had to provide more family support (e.g., elderly care), which led them to perceive more stress and was detrimental to their work [24, 25, 51]. As work plays a central role for men, they tend to develop a series of strategies to protect this domain, preventing the family from invading and damaging it [52].Although the impact on men’s work has not been as great as the impact on women’s work, many fathers have taken on more childcare responsibilities and there have also been more opportunities for work interruptions as the whole family has been at home [50]. It is psychologically damaging when the role that is most important to the person is threatened [53] (e.g., violated from another domain), which leads us expect that violations of work in the family are more detrimental to women’s recovery and that violations of family at work are more detrimental to men’s recovery.

**Fig. 1:**
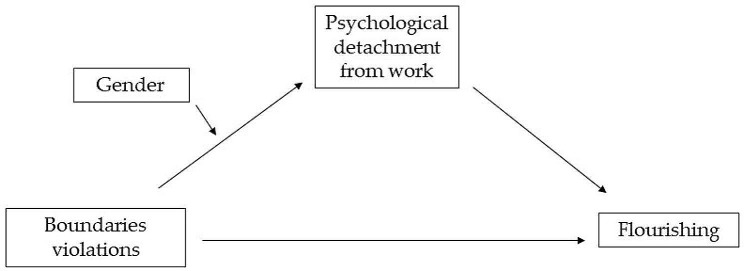
Representation of the research model

According to the stressor-detachment model [34], work stressors impair psychological detachment, which, in turn mediates the relationship between work stressors and well-being. According to role theory [47], a person invests more resources in the role that is most relevant to his/her gender (i.e., the family role tends to be valued more by women, whereas men tend to value the work role) [47, 48]. Boundary violations interfere with the psychological detachment, which, in turn, is fundamental to explain flourishing [39, 40] and this may be different for men and women. This led us to the final research model, represented in Fig. 1.

Thus, we propose a model of gender-moderated mediation of psychological detachment in the relationship between boundary violations and flourishin*g*, and suggest the following study hypotheses:

### H1a

Violations form work-to-family are negatively associated with recovery, namely, psychological detachment from work.

### H1b

Violations from family-to-work are negatively associated with recovery, namely, psychological detachment from work.

### H2a

The relationship between work-to-family violations and flourishing is mediated by recovery, namely, psychological detachment from work.

### H2b

The relationship between family-to-work violations and flourishing is mediated by recovery, namely, psychological detachment from work.

### H3a

The relationship between violations of work-to-family boundaries and recovery is moderated by gender, with this relationship being stronger in women than in men.

### H3b

The relationship between violations of family-work boundaries and recovery is moderated by gender, with this relationship being stronger in men than in women.

## Methods

### Data

Data were collected from a sample of university teachers in June 2020 in the state of Rio Grande do Sul, Brazil, during the period of mandatory confinement imposed by COVID-19. After obtaining the approval of the ethics committees of the Faculty of Passo Fundo (Brazil) (May 28th, 2020) and of the Faculty of Psychology, University of Lisbon (Portugal) (April 16th, 2020), the study was subsequently presented to the Union of Private Education Teachers of Rio Grande do Sul– Sinpro/RS, which randomly distributed the survey among 1500 members. Of these, 921 completed the questionnaires, for a 61.4% return rate. The distribution of the study and survey was conducted by email and consisted of the following components: explanation of the purpose of the study; guarantee of the anonymity of the participants; access to a *link* to the questionnaire through the *Survey Monkey* platform; and the availability of the results obtained. Informed consent was obtained in writing on the first page of the questionnaire. Participants who agreed to participate in the study proceeded to answer the questionnaire. Each participant was assigned an individual code to be entered when filling out the questionnaire, which would later allow them to obtain their results in terms of measures of stress and malaise. These individual results were included in the overall results.

### Participants

The participants were Brazilian university professors who were in mandatory telecommuting. The mean age was 44 years, and most participants were female (73.9%), married (72.3%) and had children (65.7%). A significant percentage had spouses who were also telecommuting and had children under 10 years of age (43.7% and 45.2%, respectively). None of the participants had previous experience with telecommuting, but the sample had a mean of 13.5 years of experience as teachers. More than half worked fulltime (53.5%), and the majority had an open-ended contract (86.6%) and did not perform any management or coordination role (80.1%).

### Instruments

The scales were adapted to Brazilian Portuguese by one of the authors and then verified in a pretest that was applied to 10 Brazilian university teachers. All scales are 5-point Likert scales ranging from “strongly disagree” (1) to “strongly agree” (5).

#### Boundary violations

Boundary violations were assessed using the scale of Hunter et al. [54], the original scale has an α = 0.95 in both directions (i.e., violations from work-to-family and from family-to-work), which, although not yet validated for the Portuguese population, has been previously used in a previous study in Portugal, with an α = 0.95 in violations from work-to-family and an α = 0.91 in violations from family-to-work α = 0.84 [20]. The scale allows for the evaluation of violations of borders in both directions (i.e., family-to-work and work-to-family), with each direction being evaluated through 3 items for family-to-work direction with an α = 0.79 (e.g., “*A family member has interrupted my work more than I wanted*”), and work-to-family direction, with an α = 0.88 (e.g., “My *work has interrupted my personal/family life more than I wanted to*”).

#### Psychological detachment from work

Psychological detachment from work was assessed with 3 items (e.g., “*I have had times when I distanced myself from work*”) with an α = 0.90, from the adaptation to Portuguese with an α = 0.76 [55] of the Recovery *Experiences Questionnaire* with an α = 0.90 [31].

#### Flourishing

Flourishing was assessed using the Portuguese adaptation by Silva and Caetano, with an α = 0.83 [56] of the flourishing scale of Diener et al., with an α = 0.80 [57]. For the present study, 8 items were used that evaluated the self-perception of success in relevant areas of the subject’s life (e.g., “*I lead a life with purpose and meaning*”), with an α = 0.89.

#### Gender

Participants were asked to indicate the gender with which they identified (0 = male; 1 = female).

#### Control variables

The age of the children was used as a control variable, as it has an impact on adaptation to mandatory telecommuting (1 = under 1 year old; 2 = between 1 and 5 years old; 3 = between 6 and 10 years old; 4 = between 11 and 15 years old; 5 = between 15 and 18 years old; 6 = over 18 years old).

### Data analysis

Data were analyzed with SPSS 20.0 (SPSS, 2011) and Mplus 7.0 [58]. First, we performed descriptive variable and correlation analyses with SPSS. We used Confirmatory Factor Analysis to evaluate the latent structure of the measures included in the study by Mplus 7.0. We follow the recommendation of Podsakoff et al. [59] to test the common error variance method, applying Harman’s single-factor test. Then, the best-fit model was used to evaluate the mediation model and invariance across genders. To test Hypotheses 1 and 2, that is, the mediation model, we compared models based on chi-square difference tests and on other fit indices: the standardized root mean square (SRMR), the incremental fit index (IFI), the Bentler comparative fit index (CFI) and the root mean square error of approximation (RMSEA). Regarding CFI and IFI, values greater than 0.90 represent a good model fit, and for SRMR and RMSEA, values less than 0.07 indicate a good model fit.

To test Hypotheses 3, we performed MULTIGROUP analysis. The following steps were conducted: (1) an unconstrained multiple-group model across gender, with all free parameters (baseline model), was investigated in the first step; (2) a constrained multiple-group model, where all structural paths were constrained to be equal across groups; and (3) semirestricted models that constrained the strength of different paths successively to be equal in view of inspecting invariance across the subsamples and compared these with the constraint-free models through the chi-square differences. These analyses were conducted using maximum likelihood ratio (MLR). Referring to the asymmetrical distribution between men and women, the MLR estimator was used (maximum likelihood parameter estimates with standard errors and a chi-square test statistic that are robust to nonnormality). Thus, the comparison between two nested models was tested through the significance of the difference in the chi-square value using the MLR estimator and the Satorra–Bentler scaled (mean-adjusted) chi-square.

## Results

### Descriptive variables and correlations

By means of a correlation matrix (*r*), the variables were observed to correlate with each other (*p* <.001) and are described in Table 1. All variables included in our hypotheses are correlated in the expected direction.

**Table 1:** Means, correlations and standard deviations, *n* = 921

		Mean	DP	Skewness	Kurtosis	1	2	3	4	5
1	VWF	3.13	1.17	− 0.25	− 0.91					
2	VFW	2.6	1.03	0.33	− 0.53	0.63**				
3	PDFW	2.8	1	− 0.2	− 0.92	− 0.48**	0.28**			
4	Flourishing	3.9	0.66	− 0.85	1.8	− 0.31**	− 0.26**	0.26**		
5	Gender			2.1	9.71	− 0.07*	− 0.01	0.12**	− 0.09**	
6	AYS	3.9	1.7	0.80	2.3	− 0.21**	− 0.29**	0.17**	0.14**	− 0.02

Notes: ** *p* <.01, * *p* <.05, VWF = violations from work to family; VFW = violations from family to work; WFB = work–family balance; PDFW = psychological detachment from work; Gender = 1 = male, 2 = female: AYS = age younger son

### Confirmatory factor analysis

The measurement model was constructed, including the four distinct latent constructs (work to family, family to work violations, psychological detachment from work and flourishing). The fit indices for this model showed very good fit, χ ^2^ (112) = 544.57, *p* <.01; CFI = 96; TLI = 0.95; SRMR = 0.05; RMSEA = 0.07. Then, a one-factor model was performed. The one-factor model presented poor fit to the data χ ^2^ (118) = 4607.89, *p* <.01; IFC = 54; TLI = 0.47; SRMR = 0.15; RMSEA = 0.20. Thus, our theoretical model was better than the one-factor model, Δχ ^2^ (6) = 4063.32, *p* <.01, and confirmed the construct validity of the measurement model.

### Structural equation model

To test Hypotheses 1 and 2, we performed a structural equation model in MPLUS. This model presented a very good fit (χ ^2^ (138) = 614.57, *p* <.01; CFI = 95; TLI = 0.94; SRMR = 0.05; RMSEA = 0.06). Regarding H1a, where we postulated that violations from work to the family are negatively associated with psychological detachment from work, the data indicate that the relationship was negative and significant (β= − 0.38, *p* <.01); thus, H1a was supported. H1b, where violations from family to work are negatively associated with psychological detachment from work, was also supported since this relationship was negative and significant (β= − 0.26, *p* <.01).

H2 stated that psychological detachment from work was the mediator between work-family violations and flourishing. First, was observed that the relationship between psychological detachment from work and flourishing was positive and significant (β = 0.27, *p* <.01). Second, both indirect effects were significant (violations from work to family (b = − 0.10, *p* <.01); violations from family to work (b = − 0.07, *p* <.01). However, the relationship between work-family violations and flourishing was nonsignificant (β= − 0.03, *p* >.01). Taken together, H2a was supported, and H2b was partially supported because the direct relationship between violations from family to work and flourishing was significant with the presence of a mediator (psychological detachment from work), showing that it was a partial mediation.

Regarding the control variable, the son’s age has a significant effect on psychological detachment from work (b = 0.19, *p* <.05).

### Multigroup Structural Equation Modeling (MGSEM)

The fit indices for the unrestricted (baseline) model indicated a good fit (χ ^2^ (302) = 767.41, *p* <.01; CFI = 94; TLI = 0.93; SRMR = 0.06; RMSEA = 0.07) and are described in Table 2. Following step 2, we tested the fully constrained model, and the fit indices are χ ^2^ (315) = 785,019, *p* <.01; CFI = 94; TLI = 0.93; SRMR = 0.06; RMSEA = 0.07. The chi-square difference between the unconstrained and fully constrained models was nonsignificant Δχ ^2^ (13) = 15.48, *p* >.05. Despite this nonsignificant difference, we pursued testing potential differences in the different paths.

The semirestricted Model 1a, where the relationship between violations from work to family and psychological detachment from work was constrained, presents a good fit (χ ^2^ (303) = 773,180, *p* <.01; CFI = 94; TLI = 0.93; SRMR = 0.06; RMSEA = 0.06). The difference between the unrestricted model and the semirestricted Model 1a was Δχ ^2^ (1) = 6.87, *p* <.01 and confirmed the difference between men and women in this path. The semirestricted Model 1b, where the relationship between family-to-work violations and psychological detachment from work was constrained, presents a good fit (χ ^2^ (303) = 771,567, *p* <.01; CFI = 94; TLI = 0.93; SRMR = 0.07; RMSEA = 0.06). The difference between the unrestricted model and the semirestricted Model 1b was Δχ ^2^ (1) = 6.13, *p* <.01 and confirmed the difference between men and women in this path.

**Table 2:** Model fit indices for multigroup structural equation modeling

Models	χ^2^(df)	CFI	TLI	RMSEA	SRMR	Δχ ^2^(df)
Baseline model	767.41, *p* <.01 (302)	0.94	0.93	0.07	0.06	
Constrained multiple-group	785,019, *p* <.01 (315)	0.94	0.93	0.06	0.06	15.48, *p* >.05 (13)^1^
Semi-restricted model 1a	773,180, *p* <.01 (303)	0.94	0.93	0.06	0.06	6.87, *p* <.01 (1)^2^
Semi-restricted model 1b	771,567, *p* <.01 (303)	0.94	0.93	0.06	0.07	6.13, *p* <.01 (1)^3^

Notes: baseline model = unrestricted model across gender; constrained multi-goup = a model with all structural paths were constrained to be equal across men and women; Semi-restricted model 1a = model with the relationship between violations from work to family and psychological detachment from work constrained; Semi-restricted model 1b = model with the relationship between family-to-work violations and psychological detachment from work constrained; *df* = degrees of freedom, CFI = Comparative Fit Index, TLI = Tucker–Lewis Index, RMSEA = Root Mean Square Error Of Approximation, SRMR = standardized Root Mean Square Residual; Δχ 2 = χ2—difference tests; ^1^difference between the baseline and fully constrained models; ^2^difference between the baseline model and the semi-restricted Model 1a; ^3^difference between the baseline model and the semi-restricted Model 1b

Overall, male and female differences were revealed in the relationship between work-to-family and family-to-work violations and psychological detachment from work. Specifically, violations from work to family presented a negative and significant relationship with psychological detachment from work for women (β= − 0.43, *p* <.01), while for men, this relationship was nonsignificant (β = − 0.02, *p* >.01). Furthermore, despite violations from family to work present a negative and significant relationship for women (β= − 0.20, *p* <.01) for men this relationship is stronger (β = − 0.62, *p* <.05). Thus, H3a and H3b were supported.

Regarding control variables, we observe that son’s age maintains a negative and significant effect (β = − 0.25, *p* <.01) on women’s psychological detachment, but for men, the effect was not significant (β = 0.00).

## Discussion

Based on the boundary theory [12, 13], it was observed, as expected, that boundary violations impair recovery, and that recovery mediates the relationship between violations and flourishing. Based on role theory [47], was also observed that violations from work-to-family hinder recovery more for women compared to men, and violations from family-to-work affect recovery more intensely for men.

As expected, our study indicates that boundary violations are an impediment to the recovery of university teachers who were telecommuting in mandatory confinement during COVID-19. This is an important fact, as it can be an example of the additional difficulties that arise in situations of health crisis. When university teachers had their professional activities interrupted by the family, it affected their recovery (e.g., they had to help their children with a school activity, they probably had to extend their working hours to complete all their tasks) [38]. Similarly, when university teachers saw their family lives disturbed by work (e.g., interrupting a family task to answer a call from a colleague) they probably kept thinking about work, which hindered their recovery or ability to psychologically distance themselves from work.

Thus, boundary violations can be considered to impair psychological detachment, which is most likely because they keep work mentally present or because they reduce the time allocated to leisure and rest activities that allow recovery from the expenditure of resources [22, 32, 33]. Other studies have shown that university teachers tend to keep their work psychologically present and to work outside working hours, thus presenting great difficulty in recovering [37]. As expected, it was also found that boundary violations are stressors that affected the well-being of telecommuting university teachers during mandatory confinement [33, 45, 46]. More interestingly, the results of this study also supported the assumption that the absence of recovery was the explanatory mechanism of the effect of boundary violations on well-being, because they consume resources and do not allow teleworkers to recover, which does not allow for the replacement of resources and negatively affect well-being [34].

However, we found differences according to the direction of the violation: in work-to-family violations, the relationship with flourishing is fully explained by recovery, but in family-to-work violations, in addition to this mediating effect of recovery, there is a direct relationship with flourishing. This difference may have occurred because the data collection occurred during confinement, a period in which the whole family was forced to stay at home, making this the stage for school, work and play activities, increasing stress, namely boundaries violations, especially involving the family at work [7, 22, 48]. Thus, university teachers felt that their professional activity and the quality of their teaching were affected (e.g., when they established a more distant relationship with students, had less ability to prepare classes or lacked mastery in the use of technological tools), and they felt less involvement and flourishing [41, 42].

Based on role theory [47], we observed in university teachers that violations of work in the family are more harmful to the recovery of female teachers than to male. When the family role of women is violated, they tend to invest more in family life to mitigate the negative effects of this violation, which does not allow them time to recover. These results agree with Hartig et al. [50], who emphasized that women have greater difficulty recovering when they work in the space dedicated to the family, because women attach more importance to their family role. Thus, the fact that women mostly assumed the family role increased the number of violations from work-to-family, which adversely affected their recovery [20, 22]. The weaker relationship for men results from the centrality of work role [47] and people who have work as a central role do not feel the effects of work-family violations as much [52].

Additionally, as expected, our results showed that family-work violations impair recovery, especially in the case of male university teachers. People tend to develop strategies to protect the domain that is most relevant to them [13], thus men try to prevent the family from interfering and damaging their work role. However, during mandatory telework, family violations at work were very frequent and people had been great difficulty in maintaining their work preservation strategies and great difficulty to disconnect from work, particularly those that have work as their central role.

Our study has some limitations that should be considered. First, the sample collection was performed during the first mandatory confinement, which facilitated the occurrence of boundaries violations. Thus, these data should be interpreted with caution. Despite that, data research conducted during the COVID-19 pandemic serves as a valuable resource for informing future crisis preparedness, response, and recovery efforts. By leveraging the implications of this study namely the effects of boundary violations on psychological detachment and well-being, the global community can better prepare for and mitigate the impact of future crises. Second, there was no consideration of other non-binary options, which may have had an impact on some participants’ response options. Future studies should include this option. Third, because this was a cross-sectional study, we were unable to assess causal relationships among the variables. Therefore, we recommend conducting a longitudinal study to assess causal relationships. Fourth, information on the composition of the original sample was not available to us. Fifth, we don’t know if the data was collected during the evaluation period. Future studies with university professors should take this into account. Sixth, the study uses a sample consisting of people of the same nationality, so it will be relevant to explore samples from other countries and cultures, which may differ from this sample in gender issues and the relationship between work and family. At last, it is also important to note that the participants’ technological or pedagogical skills to perform remotely tasks were not controlled. This variable could influence the relationship between variables included in this research. Thus, for future studies involving teleworkers and, specifically, university teachers, we recommend collecting participants’ technological and pedagogical skills. Future investigations should also test this model in other professions, as well as in no mandatory telework, to compare the results.

Although this study was conducted during mandatory confinement, our results suggest ways in which telecommuting should be managed to maintain or promote the well-being of teleworkers. Considering that telework is a form of work that will prevail in the future and that it facilitates violations of borders, teleworkers should use strategies that prevent the occurrence of violations, such as turning off notifications from work devices during the time dedicated to the family, limiting the period during work hours in which they can be contacted, or after the occurrence of a violation, communicate it immediately to prevent repetition [18]. To psychologically detachment themselves from work, teleworkers may use strategies such as complying with work hours, seeking to have an exclusive physical space for work, using breaks as a time to disconnect and seeking to define a routine that allows them to mentally disconnect from work, such as changing clothes and going for a walk. In addition, this study highlighted the differences between genders in the repercussions of border violations on the recovery of teleworkers. Considering that telecommuting can intensify the differences between men and women in the work-family relationship, it is important that organizations implement strategies that promote gender equality. Finally, we emphasize the social need for a more equitable distribution of family support between genders.

## Conclusion

In conclusion, this study shows that boundary violations are detrimental to the well-being of university teacher during mandatory telework, and the results of this study also support the assumption that the lack of recovery is the explanatory mechanism for the effect of boundary violations on well-being. Finally, this study also showed that work boundary violations are more detrimental to the recovery of female university teachers, whereas work boundary violations are more detrimental to the recovery of male university teachers.

## Data Availability

The datasets generated and/or analysed during the current study are available in the Figshare repository, [10.6084/m9.figshare.24243307].
